# Histone acetylation determines transcription of atypical protein kinases in rat neurons

**DOI:** 10.1038/s41598-019-40823-z

**Published:** 2019-03-13

**Authors:** Anastasia A. Borodinova, Maria A. Kuznetsova, Victoria S. Alekseeva, Pavel M. Balaban

**Affiliations:** 1 Lab of Molecular Neurobiology, Institute of Higher Nervous Activity and Neurophysiology of RAS, 5A Butlerova st, Moscow, 117485 Russia; 2 Lab of Cellular Neurobiology of Learning, Institute of Higher Nervous Activity and Neurophysiology of RAS, 5A Butlerova st, Moscow, 117485 Russia

## Abstract

It is widely accepted that memory consolidation requires *de-novo* transcription of memory-related genes. Epigenetic modifications, particularly histone acetylation, may facilitate gene transcription, but their potential molecular targets are poorly characterized. In the current study, we addressed the question of epigenetic control of atypical protein kinases (aPKC) that are critically involved in memory consolidation and maintenance. We examined the patterns of expression of two aPKC genes (*Prkci* and *Prkcz*) in rat cultured cortical neurons treated with histone deacetylase inhibitors. Histone hyperacetylation in the promoter region of *Prkci* gene elicited direct activation of transcriptional machinery, resulting in increased production of PKCλ mRNA. In parallel, histone hyperacetylation in the upstream promoter of *Prkcz* gene led to appearance of the corresponding PKCζ transcripts that are almost absent in the brain in resting conditions. In contrast, histone hyperacetylation in the downstream promoter of *Prkcz* gene was accompanied by a decreased expression of the brain-specific PKMζ products. We showed that epigenetically-triggered differential expression of PKMζ and PKCζ mRNA depended on protein synthesis. Summarizing, our results suggest that genes, encoding memory-related aPKC, may represent the molecular targets for epigenetic regulation through posttranslational histone modifications.

## Introduction

The nature of the molecular basis of memory remains a challenging problem in neurobiology. Many scientists agree that memories are stored by alterations in the strength of neuronal connections with adequate molecular machinery located in synapses. Because the inability to store information by nerve cells underlies the pathogeneses of different cognitive disorders, the search for specific molecular targets is a promising avenue for correction of memory-related pathologies. Different synaptic and nuclear molecular mechanisms work in concert to provide consolidation and reconsolidation of memory^1–6^. Experimental data suggest the existence of critical time windows, during which transient (labile) functional changes may be converted to persistent (stable) long-term memories^7–12^. These transformations, at least partially, were attributed to gene expression, since blockade of transcription during critical periods of plasticity disrupts memory consolidation in mammals and invertebrates^9,11,12^.

Transcriptional activity of genes is tightly correlated with the structural organization of chromatin: actively transcribed genomic regions are characterized by open chromatin conformation that allows the transcription factors (TFs) to interact with regulatory elements of target genes, whereas condensed chromatin interferes with the DNA-TFs interactions, thereby promoting gene silencing^13,14^. It was convincingly shown that changes in chromatin compaction can be achieved through diverse posttranslational histone modifications, especially acetylation, and this regulatory mechanism plays a crucial role in memory formation and maintenance^3,6,14,15^. In support of this, after animals are trained in specific learning paradigms, the unique patterns of histone acetylation was found in specific and expected brain areas^16–18^. Working together, the important chromatin modifiers, histone acetyltransferases (HAT) and histone deacetylases (HDAC), form the specific epigenetic landscape required for expression of diverse memory-related genes. Using a CBP mutant mice (CBP_HAT-_), lacking intrinsic histone acetyltransferase activity of CBP (CREB binding protein), Korzus and colleagues have demonstrated the critical role of histone acetylation in stabilization of long-term memories^19^. It was repeatedly shown that application of nonselective HDAC inhibitors shifts the balance to elevation of histone acetylation and promotes memory enhancement in mammals and invertebrates^5,16,19–25^, whereas active histone deacetylation facilitates memory erasure and contributes to impaired learning^26–29^. Thus, a shifted histone acetylation/deacetylation ratio in specific brain areas during critical periods of plasticity may trigger chromatin remodeling that eventually determines the expression of memory-permissive and memory-restrictive genes, therefore keeping the balance between memory maintenance and memory extinction in a transcription-dependent manner.

Over the past years, the two genes encoding atypical protein kinases (aPKC) have attracted the attention of investigators, since their protein products represent selective and accurate “molecular” tools for regulation of synaptic plasticity and memory^30–40^. Protein kinase Cλ (PKCλ), a member of the aPKC family transcribed from the *Prkci* gene, was shown to be involved in the formation of transient early memories^39^, while an intriguing brain-specific protein kinase Mζ (PKMζ), transcribed from the *Prkcz* gene^41–43^, was shown to be necessary and sufficient for long-term memory maintenance^30,31,39^. According to published data, PKMζ controls AMPA receptor trafficking to the postsynaptic membrane that may underlie the long-term enhancement of synaptic efficiency and memory maintenance^34,44–46^. The protein levels of constitutively active PKMζ increased in certain brain areas during learning, and local inhibition of the aPKC activity selectively disrupted specific memory traces^32–34,38^, without affecting the ability to re-encode the same type of memory trace again or to consolidate new memories^47,48^. Remarkably, the alternative product of the *Prkcz* gene, protein kinase Cζ (PKCζ), was actively produced in periphery organs, but was present only in trace amounts in the brain^42,49^.

The molecular organization of the 5′UTR of PKMζ transcripts restricts their translation in resting conditions^50^. However, neuronal activation helps to overcome the translational block and triggers local translation of preexisting PKMζ transcripts at the active synaptic sites. Fast synthesis and accumulation of PKMζ proteins, observed in some experiments, provides support for the translation rather than transcription as the rate-limiting step of PKMζ turnover^51^. This may be the reason why the contribution of transcription of PKMζ was underestimated for a long time. However, a recent study revealed that contextual fear learning selectively stimulates both transcription and translation of PKMζ in the prelimbic cortex of rats. The observed changes were associated with substantially decreased DNA methylation levels in the downstream *Prkcz* promoter, responsible for PKMζ synthesis^52^. These data encouraged us to investigate the potential role of epigenetic mechanisms in regulation of aPKC expression.

In the current study, using rat primary neuron cultures as a simple model, we tested whether the transcriptional activity of aPKC genes, encoding important memory regulators, may be modified in response to elevated acetylation of histones. We found that HDAC inhibitor trichostatin A directly induces the chromatin rearrangements in promoter region of the *Prkci* gene, accompanied by elevation of PKCλ expression. In contrast, the patterns of expression of PKMζ and PKCζ, reflecting activity of alternative promoters of the *Prkcz* gene, were controlled by protein synthesis-dependent mechanisms presumably implicated in promoters’ competition.

## Results

### Histone deacetylase inhibitors induce the delayed changes in mRNA levels of brain-enriched atypical protein kinases PKMζ and PKCλ

First, we answered the question whether epigenetic rearrangements, particularly histone acetylation, may influence the transcription of genes encoding a memory-permissive atypical protein kinases (aPKC). It has been convincingly shown that two aPKC isoforms, protein kinase Cλ (PKCλ) and protein kinase Mζ (PKMζ), are enriched in the brain, while the third isoform, protein kinase Cζ (PKCζ), was found in the nervous tissue in trace amounts^42,49,53^. Thus, we sought to determine the existence of dynamic regulation of both PKCλ and PKMζ mRNA levels in cultured cortical neurons incubated for 4, 8, 19 or 48 hours with trichostatin A (TSA, 100 nM), a non-selective inhibitor of histone deacetylases (HDAC).

**Figure 1:**
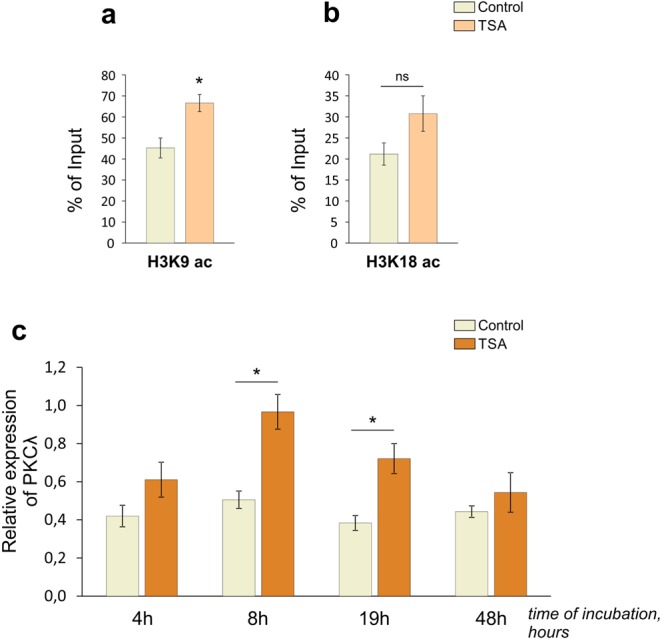
TSA slightly stimulated histone acetylation in promoter region of *Prkci* gene and upregulated PKCλ expression in cultured cortical neurons. (**a**,**b**) Figures illustrate the enrichment of acetylated histone marks H3K9 (**a**) [one-way ANOVA, F_(1,6)_ = 11,57; p = 0,0014; n = 4/group] and H3K18 (**b**) [F_(1,6)_ = 3,735; ns, p = 0,101; n = 4/group] in promoter region of *Prkci* gene in neuron cultures incubated with TSA for 19 h. Values are presented as % of Input (total amount). (**c**) Experimental timeline for detection of PKCλ mRNA levels in control cultures and cultures incubated with trichostatin A (TSA, 100 nM) for indicated time (hours). Gene expression was determined using a qPCR method and normalized to YWHAZ, encoded by *Ywhaz* housekeeping gene. [F_(7,33)_ = 7,622; p < 0,001; n = 3–8/group]. *Significant differences; ns – not significant.

Using chromatin immunoprecipitation assays (ChIP), we observed that histone acetylation levels in the promoter region of *Prkci* gene were substantially elevated in resting conditions (Fig. 1a; Supplementary Fig. S1a). Application of TSA significantly increased the quantity of particular histone modifications in the observed genome region at later time points (19 h) (Fig. 1a,b). Observed chromatin rearrangements were sufficient to induce the significant activation of PKCλ mRNA expression after 8 h of incubation with inhibitor, that were still present at a later time point, 19 h (Fig. 1c).

**Figure 2:**
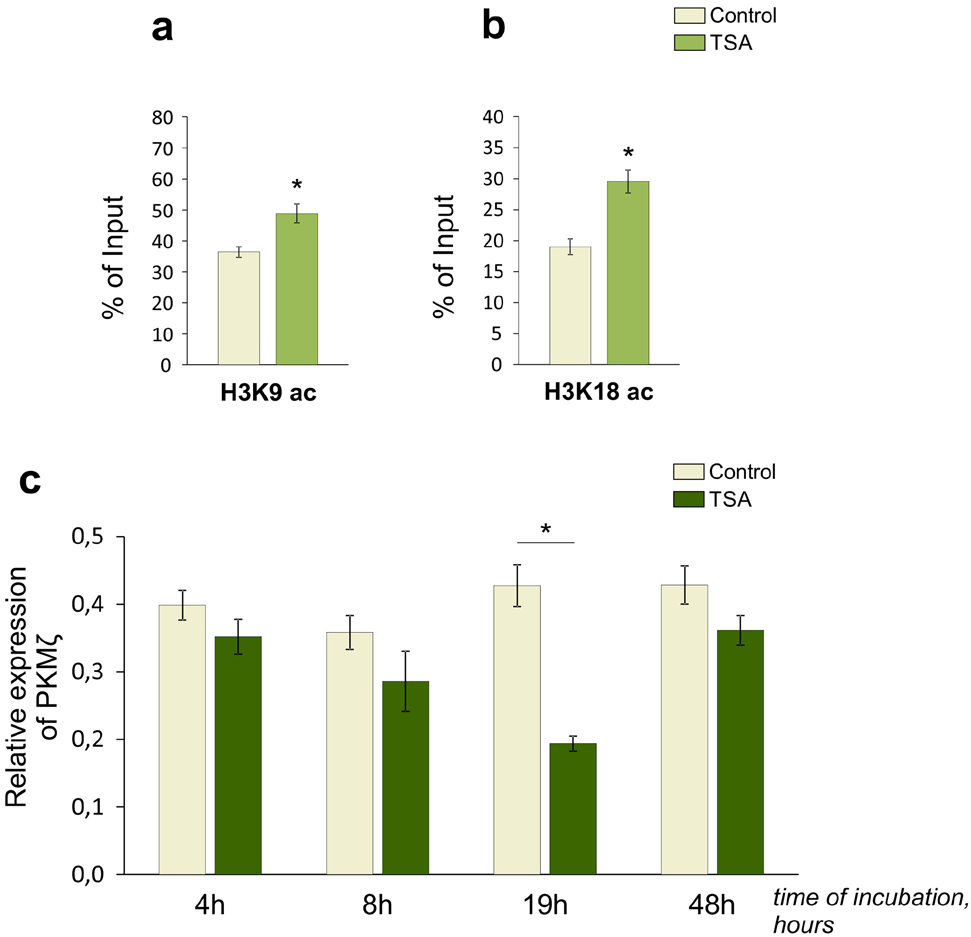
TSA stimulated acetylation of histones in promoter region of the *Prkcz* gene, responsible for PKMζ synthesis, and downregulated the PKMζ expression in cultured cortical neurons. **(a,b)** Figures illustrate the enrichment of acetylated histone marks H3K9 (**a**) [F_(1,6)_ = 12,721; p = 0,012; n = 4/group] and H3K18 (**b**) [F_(1,4)_ = 22,013; p = 0,009; n = 3/group] in promoter region of *Prkcz* gene (downstream promoter, responsible for synthesis of PKMζ transcripts) in neuron cultures incubated with TSA for 19 h. Values are presented as % of Input (total amount). **(c)** Experimental timeline for detection of PKMζ mRNA levels in control cultures and cultures incubated with trichostatin A (TSA, 100 nM) for indicated time (hours). Gene expression was determined using a qPCR method and normalized to YWHAZ [Kruskal-Wallis (KW): H (7, N = 39) = 23,01684 p = 0,0017; *p < 0,001; n = 4–6/group]. *Significant differences.

In contrast, the presence of acetylation marks in the downstream promoter of the *Prkcz* gene appeared shortly after TSA treatment (4 h, Supplementary Fig. S1b), and persisted at least during 19 h of incubation with TSA (Fig. 2a,b). However, the fast development of chromatin rearrangements in cultured cortical neurons did not induce any reliable changes in the expression of the corresponding PKMζ transcripts at the 4 h time point (Fig. 2c). Persistence of acetylated histone marks H3K9 (histone H3 lysine 9, Fig. 2a) and H3K18 (histone H3 lysine 18, Fig. 2b) in the downstream promoter of *Prkcz* gene within 19 h after TSA treatment was accompanied by a reduced expression of PKMζ in cultured cortical neurons (Fig. 2c). This was surprising because histone acetylation was usually associated with facilitation of transcription. However, downregulation of gene expression, induced by HDAC inhibitors, has been previously described for some genes^54,55^.

To exclude the possibility that the TSA-induced changes in expression of aPKC were associated with delayed nonspecific effects of the inhibitor^56^, we performed a similar experiment with alternative HDAC inhibitor sodium butyrate (NaB). Administration of NaB (5 mM) for 19 h induced a substantial elevation of PKCλ expression (Supplementary Fig. S1c) that was consistent with our results (Fig. 1c), and with the previously published data, describing the elevation of PKCλ expression in the rat cortical cultures incubated with HDAC inhibitor valproic acid for 12 h^55^. Comparison of the experimental results between NaB-treated (Supplementary Fig. S1d) and TSA-treated groups (Fig. 2c) revealed identical impairments of the PKMζ expression. Together, these data indicate that histone acetylation level is important for the regulation of the aPKC expression patterns in the primary neuron cultures, at least in basal conditions.

### Expression of predominantly peripheral atypical protein kinase Cζ appeared in cultured cortical neurons after treatment with histone deacetylase inhibitors

**Figure 3:**
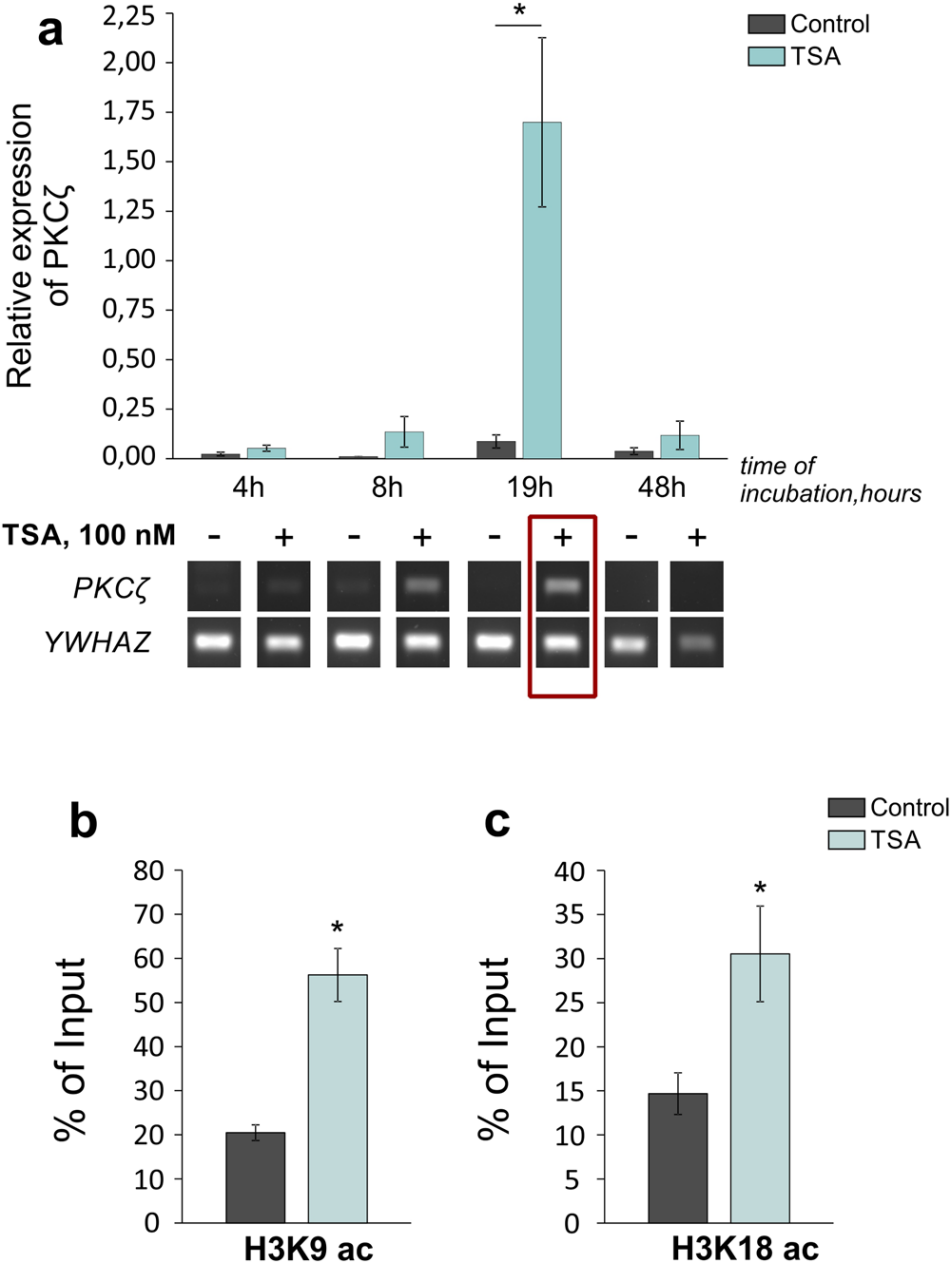
TSA substantially increased acetylation of histones in promoter region of *Prkcz* gene, responsible for PKCζ synthesis, and upregulated PKCζ expression in cultured cortical neurons. **(a)** Experimental timeline for detection of PKCζ mRNA levels in control cultures and cultures incubated with 100 nM TSA for indicated time (hours) [KW: H (7, N = 38) = 21,64737 p = 0,0029; *p < 0,001; n = 3–8/group]. Representative experiment below demonstrates the dynamic changes of PKCζ expression in control cultures and cultures incubated with trichostatin A (TSA, 100 nM) for indicated time (hours) determined by standard PCR followed by agarose gel electrophoresis. YWHAZ served as a reference. Red rectangle marks the most prominent changes in mRNA level of PKCζ. **(b,c)** Figures illustrate the enrichment of acetylated histone modifications H3K9 (**b**) [F_(1,6)_ = 32,458; p < 0,001; n = 4/group] and H3K18 (**c**) [F_(1,4)_ = 10,086; p = 0,034; n = 3/group] in promoter region of *Prkcz* gene (upstream promoter, responsible for synthesis of PKCζ transcripts) in neuron cultures incubated with TSA for 19 h. Values are presented as % of Input (total amount). *Significant differences.

According to the experimental data, the PKCζ isoform is found in mammalian nervous tissues in trace amounts, however it is widely present in different periphery organs^42,49^. This strongly suggests the existence of tissue-specific regulation of the PKCζ expression in mammals, which, in our opinion, may be determined by a certain epigenetic status in the particular cell types. Our results were consistent with the previously published data, demonstrating the barely detectable levels of PKCζ mRNA in control cultures of cortical neurons (Fig. 3a; Supplementary Fig. S2a). The levels of histone acetylation in the upstream *Prkcz* promoter, responsible for the PKCζ synthesis, was low in resting conditions (Fig. 3b,c; Supplementary Fig. S2b).

We wondered whether chromatin rearrangements, similar to those observed during memory formation, may remove the transcriptional silencing and facilitate PKCζ expression. To verify the possibility of epigenetic regulation of PKCζ expression in nervous tissue, we treated cultured cortical neurons with HDAC inhibitors to induce massive histone acetylation similar to the one observed during learning^16–18^. Our results suggest that short treatment (4 h) of cultures with TSA (Supplementary Fig. S2b) produced substantial enhancement of the histone acetylation marks in upstream promoter of *Prkcz* gene that persisted at least over 19 h (Fig. 3b,c). In parallel, we registered the delayed elevation (approximately 18-fold) of the PKCζ transcript quantities in response to continuous incubation of cultures with either TSA (Fig. 3a) or NaB (Supplementary Fig. S2a). Summarizing, our results support the idea of tissue-specific regulation of the PKCζ transcription, and propose this protein kinase as one of the epigenetic targets.

### Histone deacetylase inhibitor trichostatin A alters the mRNA repertoire of the atypical protein kinases in a transcription-dependent manner

**Figure 4:**
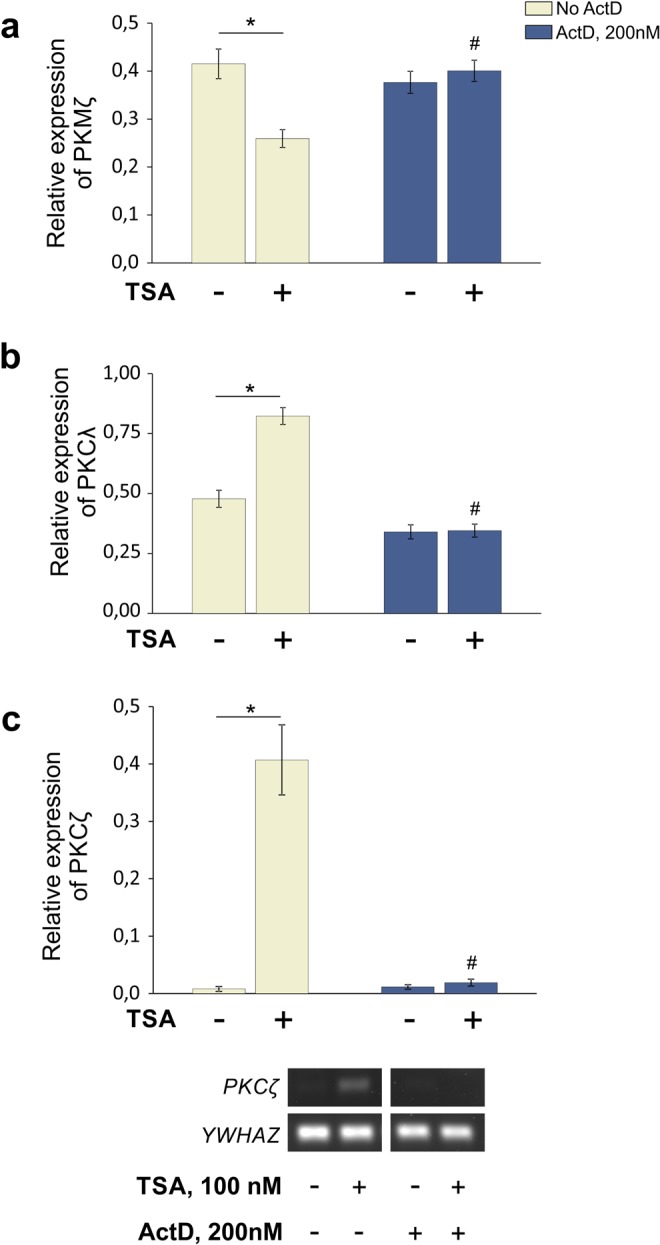
Transcriptional blockade with actinomycin D (ActD, 200 nM) prevented TSA-induced changes in mRNA levels of aPKC. qPCR analysis of PKMζ (**a**) and PKCλ (**b**) gene expression. Control, TSA-treated cultures (19 h), ActD-treated cultures (20 h) and cultures pretreated for 1 h with ActD followed by 19 h of incubation with TSA (20 h in sum) were used for comparisons. [KW: H (3, N = 16) = 8,161765 p = 0,0428; *p = 0,003; ^#^p = 0,005; *n* = *4/group* for PKMζ], [F_(3,15)_ = 45,383; p < 0,001; n = 4–5/group for PKCλ]. (**c**) qPCR analysis of PKCζ gene expression [KW: H (3, N = 22) = 13,76000 p = 0,0033; *p < 0,001; ^#^p < 0,001; n = 5–6/group]. Representative experiment below demonstrates the changes of PKCζ mRNA levels in four experimental groups (see above) determined by standard PCR followed by agarose gel electrophoresis. YWHAZ served as a reference. *Significant differences; ^#^Significant difference (relative to standard TSA-treated group).

Histone acetylation promotes chromatin rearrangements, which usually facilitate gene transcription and subsequent accumulation of the newly generated mRNA. Therefore, an observed elevation of PKCλ and PKCζ mRNA quantities after administration of HDAC inhibitors presumably can be attributed to the direct transcriptional activation caused by chromatin rearrangements. However, the decreased number of PKMζ transcripts observed in our experiments may suggest either an epigenetically triggered transcriptional silencing^55^ or a posttranscriptional mRNA destabilization^57^. To verify whether TSA directly controlled mRNA synthesis from aPKC genes, we performed a series of experiments with a nonspecific transcription blocker actinomycin D (ActD), which inhibits RNA polymerases (RNAPs). In each experiment, we had four groups of cultured neurons: a standard control, a standard TSA-treated group (19 h), an ActD-treated group, and a group treated with TSA in the presence of ActD. Neurons of the third group were incubated with ActD at a final concentration of 200 nM (Fig. 4) or 4 μM (data not shown) during 20 h. Figure 4 demonstrated only slight changes in basic levels of aPKC mRNA in cultured neurons after incubation with the inhibitor, which may be explained by the general impairment of gene transcription. The fourth experimental group was pretreated with ActD for 1 h prior to the TSA administration, and afterwards the cortical neurons were continuously incubated with both inhibitors for 19 h (in total 20 h; Fig. 4).

Our observations from these experiments confirmed our initial hypothesis of the epigenetically-driven modulation of the aPKC gene transcription. We showed that transcriptional blockade with ActD (Fig. 4a) prevented reduction in the quantity of PKMζ transcripts resulting from TSA treatment. These data presumably indicate that the observed changes of PKMζ expression can be based on the transcriptional silencing rather than mRNA degradation. Also pretreatment of the neuron cultures with ActD completely prevented activation of both PKCλ (Fig. 4b) and PKCζ expression (Fig. 4c) induced by the HDAC inhibitors. Summarizing, these lines of evidence revealed the tight interplay between histone acetylation and the activity of aPKC transcriptional machinery.

### Epigenetic control of *Prkcz* transcripts quantity is protein synthesis-dependent, whereas epigenetically triggered accumulation of *Prkci* transcripts is protein synthesis-independent

**Figure 5:**
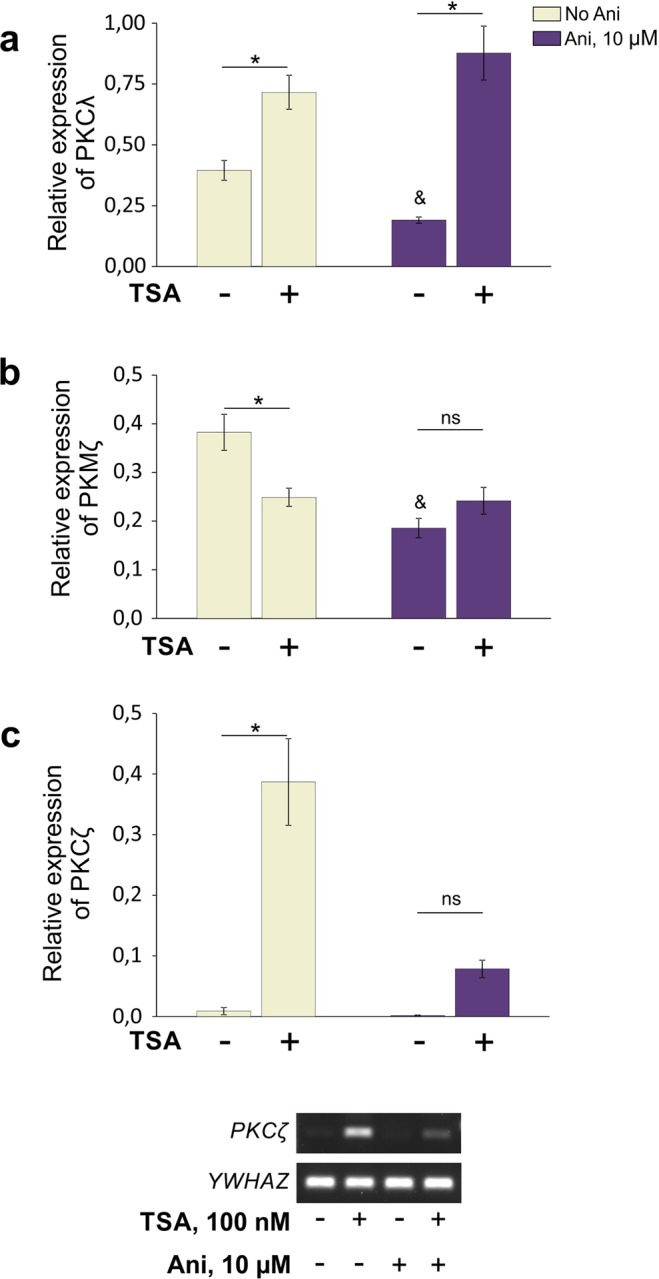
Blockade of translation with anisomycin (Ani, 10 μM) selectively altered the TSA-induced regulation of PKMζ and PKCζ expression, while epigenetically-driven upregulation of PKCλ expression was unaffected. (**a**) Application of Ani did not impair the TSA-induced upregulation of PKCλ expression. Control, TSA-treated (19 h) cultures and cultures treated with Ani alone (20 h) or in combinations with TSA were used for comparisons. For the last group, Ani was administered for 1 h before the application of TSA. [F_(3,17)_ = 20,913; p < 0,001; n = 5–6/group]. (**b**,**c**) Ani prevented downregulation of PKMζ expression in TSA-treated cultures [KW: H (3, N = 24) = 13,22000 p = 0,0042; *p = 0,002; ^&^p < 0,001; n = 6/group] and significantly alleviated epigenetically-driven upregulation of PKCζ expression [KW: H (3, N = 20) = 16,45893 p = 0,0009; *p < 0,001; n = 5/group]. Representative experiment below demonstrates patterns of PKCζ expression in the current experimental series. mRNA levels were determined by standard PCR followed by agarose gel electrophoresis. YWHAZ served as a reference. *Significant differences; ^&^Significant difference (relative to standard control group); ns – not significant.

Next, we tested whether TSA acts indirectly, inducing *de-novo* synthesis of proteins that contributed to regulation of aPKC expression. To answer this question, we performed a series of experiments with anisomycin (Ani), which blocks translation. In each experiment we operated with four groups of cultured neurons: standard control, standard TSA-treated group (19 h), Ani-treated group, and a group treated with TSA in the presence of Ani. Anisomycin in final concentration 10 μM (Fig. 5) or 100 μM (data not shown) was applied to the culture medium of the third group for 20 h, while the fourth group was subjected to pretreatment with Ani for 1 h (without washout), followed by incubation with TSA for 19 h. Subsequent analysis of quantitative real-time PCR (qPCR) data revealed two distinct mechanisms contributing to regulation of *Prkci* and *Prkcz* gene activity. Our results demonstrate that overproduction of PKCλ mRNA molecules, which was observed in response to TSA administration, persists even in the presence of anisomycin (Fig. 5a). Therefore, we concluded that transcriptional activity of *Prkci* gene may be enhanced after epigenetic rearrangements, and this upregulation is realized directly, in a protein synthesis-independent manner. In contrast, epigenetic regulation of both PKMζ and PKCζ expression was disrupted in the presence of anisomycin (Fig. 5b,c, respectively). The reasonable explanation of these results would be that the patterns of PKMζ and PKCζ expression were regulated indirectly through a protein synthesis-dependent mechanisms, which were activated in response to epigenetic rearrangements.

## Discussion

Different learning processes, such as consolidation, reconsolidation and extinction, have been shown to be accompanied by diverse region-specific chromatin rearrangements which may be implicated in regulation of synaptic plasticity and memory mechanisms^6,16–18,21,26,58–60^. The epigenetic status of chromatin plays a fundamental role in the regulation of transcriptional machinery. Groups of chromatin-modifying enzymes, such as histone deacetylases (HDAC) and histone acetyltransferases (HAT), along with the DNA-modifying enzymes, work cooperatively to provide a particular structural and functional organization of chromatin, required for activation or silencing of memory-permissive and memory-restrictive genes. The role of epigenetics in the regulation of particular memory-related genes has been reported in many previous papers^19,20,26,52,58,59,61^.

A goal of the present study was to determine whether histone acetylation controls expression of genes encoding atypical protein kinases (aPKC) in primary cultures of rat cortical neurons, which represent a simple model of neuronal networks. PKCλ and PKMζ isoforms were of particular interest, because their protein products play pivotal, temporally distinct roles in synaptic plasticity and memory mechanisms^30–32,35,36,39,40^. To influence the levels of histone acetylation, we applied the nonspecific HDAC inhibitors (TSA, NaB) that were commonly used for behavioral studies of learning and memory in vertebrates and invertebrates^5,16,19–23,25^. Our findings suggest that diverse molecular mechanisms may underlie the observed epigenetically-triggered changes in expression patterns of different aPKC.

We showed that continuous incubation of cultured neurons with TSA induced a broad histone acetylation (Fig. 1a,b), and facilitated *Prkci* gene transcription. The considerable rise of PKCλ mRNA quantities appeared relatively quickly after TSA administration (8 h) and persisted up to 19 h (Fig. 1c; Supplementary Fig. S1c). Further experiments with inhibitors of transcription (ActD; Fig. 4b) and translation (Ani; Fig. 5a) revealed that epigenetic rearrangements stimulated the *Prkci* gene transcription and accumulation of PKCλ mRNA directly in a protein synthesis-independent manner. We speculate that histone acetylation observed during learning^16–18^ may similarly activate *Prkci* gene expression and lead to accumulation of PKCλ molecules, essential for early synaptic plasticity mechanisms and short-term memory^39^. However, this requires further investigation.

We found that upstream and downstream *Prkcz* promoters respond differently to HDAC inhibitors: the expression of brain-enriched PKMζ was significantly decreased (Fig. 2c, Supplementary Fig. S1d), while the expression of PKCζ was substantially elevated (Fig. 3a; Supplementary Fig. S2a). These data provide insights into tissue-specific activity of the alternative *Prkcz* promoters and raise questions about the underlying molecular mechanisms. Our findings regarding the epigenetic regulation of *Prkcz* gene expression revealed the considerable delay between the appearance of histone acetylation marks (4 h; Supplementary Figs S1b, S2b), and the consecutive changes in expression of PKMζ and PKCζ isoforms (19 h; Figs 2c and 3a). Our results suggest the existence of an indirect epigenetic-sensitive molecular mechanism, required for regulation of the *Prkcz* gene expression^56^. To further determine the nature of this mechanism, we performed an analysis using different inhibitors that impaired the transcription (Fig. 4a,c) and translation (Fig. 5b,c). We assumed that downregulation of PKMζ expression might be achieved either through epigenetically triggered transcriptional silencing^55^, or via selective post-transcriptional mRNA destabilization and/or degradation^54,57,62^. Our experiments with transcriptional inhibitor ActD confirmed that HDAC inhibitors preferentially influenced the *Prkcz* gene transcription rather than degradation of PKMζ mRNA (Fig. 4a).

Our next experiments with translation inhibitor (Ani) revealed the most remarkable result. It turned out that the epigenetic regulation of *Prkcz* promoter’s competition, resulting in changes in expression of PKMζ and PKCζ,  was tightly associated with *de-novo* protein synthesis (Fig. 5b,c). The short lifespan (hours) of TSA reduced the time window during which the synthesis of potential protein regulators can be activated^63,64^. This may propose members from the family of immediate early genes as the most probable candidates in mediating the fine-tuned epigenetic regulation of *Prkcz* gene expression.

The *Prkcz* gene is, perhaps, the most intriguing among all genes encoding different protein kinase C isoforms. It was thought for a long time that the single “fully-equipped” PKCζ product, containing regulatory and catalytic domains, was transcribed from the *Prkcz* gene. However, the alternative downstream *Prkcz* promoter, responsible for the synthesis of the truncated constantly active PKMζ product, was discovered in the 2000’s^41,42^. At that time, the attention of researchers was focused exclusively on the brain-enriched PKMζ^42,49,53^, which was further proposed as the necessary and sufficient molecule for memory consolidation and maintenance^30,31,35,39^. In contrast, PKCζ was underrepresented in the different brain regions of mammals, which discouraged attempts to investigate the potential role of PKCζ in the mammalian brain^42,49^. Consistent with previously published data, our results confirmed the presence of PKMζ products (Fig. 2c; Supplementary Fig. S1d), and almost nonexistent PKCζ products in neurons in resting conditions (Fig. 3a; Supplementary Fig. S2a).

Mutually exclusive distribution of PKCζ and PKMζ isoforms presumably reflects the tissue-specific activities of the corresponding *Prkcz* promoters and can be determined by local specificity of transcriptional regulation and chromatin microenvironment. Our data extend these findings, providing indirect evidence of the epigenetically-driven *Prkcz* promoter competition. We found that upstream and downstream *Prkcz* promoters respond differently to HDAC inhibitors, giving the prevalence of either PKCζ or PKMζ expression in particular conditions. According to our ChIP experiments, TSA induced rapid and persistent histone acetylation in both silent upstream and active downstream promoters of *Prkcz* gene in cultured cortical neurons (Figs 2a,b; 3b,c; Supplementary Figs S1b, S2b). Unexpectedly, these changes stimulated transcription from the upstream *Prkcz* promoter, which resulted in tremendous elevation of the PKCζ mRNA quantity (Fig. 3a). Our pilot experiment showed that active production of PKCζ transcripts was accompanied by elevation of the PKCζ protein products (Supplementary Fig. S3). The importance of PKMζ for synaptic processes has been counterbalanced by the lack of evidence for a potential role of PKCζ in synaptic processes in the nervous system of mammals and invertebrates^37,65–67^. However, our results, in combination with the observation that PKCζ has been found in nuclear compartments of neurons and non-neuronal cells in different animals^68–70^, should encourage others to reconsider the functions of PKCζ in both synaptic and nuclear processes.

In parallel with active epigenetic regulation of PKCζ expression, the quantity of memory-permissive PKMζ dropped significantly in the same conditions (Fig. 2c; Supplementary Fig. S1d). As far as we know, this is the first evidence of the “natural” downregulation of PKMζ mRNA, achieved without any synthetic genetically encoded construct, or a specific learning paradigm. Interestingly, our pilot experiments demonstrated that the PKMζ protein expression was similarly decreased in response to TSA administration (Supplementary Fig. S3). Not surprisingly, these data raise questions about our observation of unusual PKMζ downregulation and its functional implications. In previous studies, the presence of PKMζ was always considered as a necessary factor for long-term synaptic potentiation, successful learning and memory consolidation^30,31,33,35,36,39,40,48,52^. On the contrary, a decreased quantity of PKMζ was associated with the long-term synaptic depression, impaired learning and weakening of the existing memory trace^32,33,35,40,47,52,71–73^. Previously, it was described in details that consolidation of some types of memory requires both long-term potentiation and long-term depression (LTD)^74^. It has been proposed that induction of LTD during learning may suppress any interference from the previously established memory traces in the same neuronal circuits, and facilitate memory updating^74^. We suggest that our manipulations of epigenetics in cultured neurons and the related transcriptional changes caused by these manipulations (Figs 2c, 3a; Supplementary Fig. S1d, S2a) may be a part of a natural LTD-like processes that, *in vivo*, are involved in either memory consolidation or memory labilization (erasure, forgetting). However, we understand that our observations may be limited in nature, because all experiments were performed using primary neuron cultures. Therefore, our results motivate further investigation in *in vivo* models.

## Materials and Methods

### Animals

Newborn Wistar rat pups (P0–P1) were used for the primary neuron cultures preparation. All experimental procedures were conducted in accordance with the European Communities Council Directive of 24 November 1986 (86/609/EEC) on the protection of animals used for scientific purposes. The study protocol was approved by the Ethics Committee of the Institute of Higher Nervous Activity and Neurophysiology of RAS.

### Primary cultures of cortical neurons

Cell cultures preparation was accomplished according to the standard protocol. Briefly, Wistar rat pups were euthanized by decapitation with sharp scissors. The brains were removed and placed in the chilled Dulbecco’s modified Eagle medium (DMEM) supplied with glutamine and glucose (Paneco), then cortices were dissected and gently cut into pieces with a sharp blade. After that, tissue was transferred to a warm DMEM solution with trypsin (10 mg in 12,5 ml of solution; MP Biomedicals) and incubated for 15 min at 37 °C. Treated tissue was centrifuged at 2000 rpm for 2 min, then washed with chilled DMEM solution to stop trypsin activity and centrifuged again at 2000 rpm for 2 min. Cell pellets were re-suspended in Neurobasal medium (Gibco) supplemented with 2% B-27 serum (Gibco) and alanyl-glutamine (Paneco). After mild trituration isolated cells were counted in Goryaev’s chamber. For qPCR experiments approximately 2–2,5 × 10^5^ cells were placed in 24-well plates onto each 12 mm glass coverslip coated with poly-D-lysine hydrobromide (Sigma). For ChIP experiments approximately 2–2,5 × 10^6^ cells were placed in 6-well plates into individual wells coated with poly-D-lysine. One hour later the culture media were replaced. Cortical cultures were grown for two weeks in CO_2_ incubator (5% CO_2_, 37 °C), culture medium was refreshed regularly. Drugs were applied on the 14^th^ or 15^th^ day *in vitro* (DIV). All experiments had at least three biological replicates.

### Drugs

Histone deacetylase inhibitors trichostatin A (TSA, final concentration 100 nM, Sigma) and sodium butyrate (NaB, final concentration 5 mM, Sigma-Aldrich) were used for investigation of the role of histone acetylation in the regulation of aPKC expression. Cultures were incubated with TSA for 4, 8, 19, 48 hours, or with NaB for 19 hours.

Transcription inhibitor actinomycin D (ActD, final concentration 200 nM, 4 μM; Sigma-Aldrich), and protein synthesis inhibitor anisomycin (Ani, final concentration 10 μM, 100 μM; Tocris) were used for verification of experiments with histone deacetylase inhibitors. Experimental cultures were pretreated with ActD or Ani for 1 hour (without washout), followed by continuous incubation with TSA (19 hours). Control cultures were incubated with ActD or Ani for 20 hours.

### mRNA extraction and qPCR analysis

Total RNA from primary neuron cultures was prepared according to manufacturer’s Extract RNA protocol (Evrogen), then aliquoted and stored at −70 °C. Equal amounts of total RNA (approximately 400–500 ng) were taken for cDNA synthesis using MMLV RT kit (Evrogen) and random decamer primers (Evrogen). Then cDNA was subjected to quantitative real-time PCR (for PKMζ, PKCλ, PKCζ) or standard PCR with subsequent analysis by electrophoresis (for PKCζ). The following pairs of primers were used for analysis of aPKC gene expression: PKMζ: forward, 5′-CTATTGTCGATCCGGAGACCCA-3′; reverse, 5′-TCTGCTGCCTCTTCAGCACGGAA-3′; PKCζ: forward, 5′-CAGGACCTCTGTGAGGAAGTGC-3′; reverse, 5′-GGTTGTTCTGGGATGCTTGGGA-3′; PKCλ: forward, 5′-GGACAATGTACTGCTGGACTCTG-3′; reverse, 5′-CTGAAGCCATAGTCTTCTCCTCT-3′. Primers specific for YWHAZ were used as a control (forward, 5′-TTGAGCAGAAGACGGAAGGT-3′; reverse, 5′-GAAGCATTGGGGATCAAGAA-3′). The PCR products of all aPKC were verified by sequencing. Briefly, aPKC fragments were amplified using respective primer pairs and cDNA template, then PCR products were ligated to the standard pTZ57R/T vector (InsTAclone PCR cloning kit; Thermo Fisher) that was used further for transformation of *E. coli* Top10 strain. The DNA plasmids from single bacterial colonies were purified (MiniPrep kit; Evrogen) and analysed by custom DNA sequencing (Evrogen).

To analyse expression of PKMζ and PKCλ, qPCR was carried out with SYBR-green mastermix reagent (Evrogen) using 7500 Real Time PCR system (Applied Biosystems, California, USA) according to the following cycling conditions: 95 °C for 5 min; 40 cycles of 95 °C for 30 s, 60 °C for 30 s and 72 °C for 30 s. For each sample, the reaction was run in triplicates in 96-well plates. To analyse expression of PKCζ, qPCR was carried out with SYBR-green mastermix reagent (Evrogen) using C1000 Touch Thermal Cycler (Bio-Rad, California, USA) according to the following cycling conditions: 95 °C for 5 min; 42 cycles of 95 °C for 30 s, 61 °C for 30 s and 72 °C for 30 s. For each sample, the reaction was run in triplicates in 384-well plates. Detection of fluorescent signals occurred at the end of each 30 s 72 °C temperature step. Values were normalised to the *Ywhaz*, relative levels of aPKC expression were calculated using standard ΔΔCt method.

Analysis of PKCζ expression was also performed using standard PCR with the same cycling conditions (see above) followed by electrophoresis in 1,5% agarose gel. YWHAZ was used as a reference control.

### Chromatin extraction

For ChIP experiments we have used cortical cultures grown in 6-well plates (approximately 2–2,5 × 10^6^ per well). Each plate contained 3 control and 3 TSA-treated wells. Cells in each well were incubated with 38% formaldehyde (Sigma) at room temperature for 10 min (formaldehyde was added directly to 2 ml of culture medium to the final concentration of 1%). DNA-proteins cross-links were quenched by adding 2.5 M glycine (to the final concentration of 0,125 M) followed by 20 min incubation at 4 °C. Next, cells were rinsed with 5 ml of cold 1x phosphate-buffered saline (PBS, Sigma), scraped from dishes thoroughly with a cell scrapers and transferred into two 15 ml tubes corresponding to control and TSA-treated groups. Cells were pelleted by centrifugation, resuspended in 1 ml of cold (PBS) and homogenized in Potter-type apparatus. Next, we washed samples twice with 10 ml of cold PBS (1×), resuspended cell pellets in 10 ml of chilled Cell Lysis buffer (5 mM HEPES, pH 8,0; 85 mM KCl; 0,5% Nonidet P-40) and incubated the samples for 10 min at 4 °C. Cell nuclei were checked in Gorjaev’s chamber and then were resuspended in 350 μL of freshly prepared Nuclei Lysis Buffer (50 mM Tris HCl, pH 8,0; 10 mM EDTA; 1% SDS) supplemented with 10X protease inhibitor cocktail (cOmplete Mini; Roche). After 10 min incubation at 4 °C lysates were sonicated in Bioruptor Sonication System (Bioruptor, Cat.# UCD-200; Diagenode) with the following settings: high setting, 45 s ON; 15 s ON, 45 s OFF for 5 min. Cell debris was pelleted by centrifugation for 15 min, 4 °C, 13,000 × g. Supernatants were transferred to a new tubes and frozen at −70 °C.

Before each immunoprecipitation (IP), an aliquot of sonicated chromatin was used to check the DNA fragments size. To remove cross-links, chromatin was incubated with 4 µL of 5 M NaCl overnight at 65 °C. On the next day we mixed samples with 2 µL 0,5 M EDTA, 4 µL 1 M TrisHCl and 0,4 µL Proteinase K (Thermo Fisher) and incubated for 1 h at 42 °C. Then DNA was purified using standard phenol:chloroform extraction and analysed by agarose gel electrophoresis. The average fragments were in 200–500 bp range.

### ChIP and qPCR

Chromatin Immunoprecipitation was performed using OneDay ChIP kit (Diagenode) as recommended in the manual with insignificant changes. Briefly, chromatin aliquots were thawed on ice, then 60 μL of each sample was incubated with either 5 µL of H3K9ac or H3K18ac antibodies (Cat.#9649 and Cat.#13998, respectively; Cell Signaling) or 1 µL of IgG antibodies (Cat.#2729, Cell Signaling) on a rotating wheel overnight at 4 °C. Chromatin from Input samples, corresponding to 10% of total amount, was precipitated with glycogen (Thermo Fisher) in 96% ethanol. Then we washed chromatin pellets with 75% ethanol and left overnight at 4 °C. On the next day, washed antibody binding beads were incubated with chromatin-antibody complexes for 30 min on a rotating wheel at 4 °C to form [chromatin-antibody-beads] complexes. Bound chromatin was washed with 1x ChIP Buffer. Then samples proceeded to the DNA purification step. Input samples were dried and diluted in PCR-grade water from the Kit. All the samples (both Input and [chromatin-antibody-beads] complexes) were mixed with DNA purifying slurry and incubated for 10 min at 99 °C. Next, samples were treated with 1 µL Proteinase K (200×, provided in kit) for 30 min at 55 °C in a thermomixer (1000 rpm), followed by heating up to 99 °C during 10 min. Supernatants, containing purified chromatin, were collected twice according to the protocol to final volume 200 µL.

Quantitative real-time PCR analysis of samples was carried out with SYBR-green mastermix reagents (Evrogen) using a 7500 Real Time PCR system (Applied Biosystems; California, USA) according to the following cycling conditions: 95 °C for 5 min; 40 cycles of 95 °C for 30 s, 62 °C for 30 s and 72 °C for 30 s. Reactions for each sample were run in triplicates in 96-well plates. Detection of fluorescent signals occurred at the end of each 30 s 72 °C temperature step.

The following primer pairs were used for analysis of promoter regions of aPKC genes: PKMζ: forward, 5′-GGAAGGACCCTAGTCACCTG-3′; reverse, 5′-ATGGATACGGAGGAGCTGAC-3′; PKCζ: forward, 5′-GAAAACACCGGACGACCCCAA-3′; reverse, 5′-CCACCTGCGGCCAATGAGAG-3′; PKCλ: forward, 5′-CCTCAGATACTCAAGCTGTTCTC-3′; reverse, 5′-CGCTCAAAGTTGTTTCCCCTAT-3′. The efficiency and specificity of primers was tested on the chromatin samples extracted from the brain tissue. Values were normalised to Input samples and enrichment was presented as % of total DNA.

### Statistical analysis

Results are presented as Mean ± SEM. Statistical significance of differences between the groups with normally distributed data was assessed with one-way ANOVA test followed by Tukey’s post hoc test (SigmaPlot v.11, Systat Software). Significance for non-normally distributed data was calculated using non-parametric Kruskal-Wallis (KW) method followed by Duncan’s post hoc test (Statistica v.10 software, StatSoft). Significance was set at p < 0,05. All experiments were performed in, at least, three biological replicas, the numbers of independent cultures in each experimental group (n) are indicated in figure legends.

## Supplementary information

Supplementary Figures and Methods
