# Supplementary material for: Histone acetylation determines transcription of atypical protein kinases in rat neurons
Source: Sci Rep. 2019 Mar 13;9:4332. doi: 10.1038/s41598-019-40823-z (PMC6416243; doi:10.1038/s41598-019-40823-z)
Supplement: Supplementary file 1 — Supplementary Figures and Methods [file 41598_2019_40823_MOESM1_ESM.pdf]

Supplementary Figures and Methods

Histone acetylation determines expression of atypical protein kinases in rat neurons

Anastasia A. Borodinova[1,*], Maria A. Kuznetsova[1], Victoria S. Alekseeva[1], Pavel M. Balaban[2]

[1]Lab of Molecular Neurobiology, Institute of Higher Nervous Activity and Neurophysiology of RAS, 5A Butlerova st, Moscow, 117485, Russia

[2]Lab of Cellular Neurobiology of Learning, Institute of Higher Nervous Activity and Neurophysiology of RAS, 5A Butlerova st, Moscow, 117485, Russia

Supplementary methods

Protein extraction

We used rat cortical cultures grown in 6-well plates for 14 days (approximately 2-2,5 x106 per well). Each well corresponded to one sample (control or treated with TSA for 19 h). The protein extraction was performed according to the standard protocol. Briefly, neurons were washed twice with cold 1x PBS (Sigma). Then neurons were scraped from dishes thoroughly with a cell scrapers in 1ml of PBS supplemented with 10X protease inhibitor cocktail (PIC, cOmplete Mini; Roche). After centrifugation (2500 x g, 5 min, 4°C) a supernatants were discarded. Cell pellets were resuspended in 60 µl of 1x RIPA buffer with 10x PIC, prepared according to the manufacturer's protocol (Thermo Fisher). The mixture was incubated on ice at constant shaking for 15 minutes and then centrifuged (14 000 x g, 15 min, 4°C). The supernatants were transferred in the new tubes. The protein concentrations in the samples were determined using Pierce BCA Protein Assay kit (Thermo Fisher). Absorbance of the calibration and experimental samples was measured in the plate reader Infinite M200 Pro (Tecan, Switzerland). Then samples were aliquoted and frozen at -70°C for further use.

Western blotting.

Western blotting method was used to uncover whether the changes of *Prkcz* gene transcription were also expressed at the protein levels.

We used the TGX Stain-Free FastCast Acrylamide kit, 12% (Bio-Rad, USA) to prepare the 75 mm-thick gels for electrophoresis. For one reaction we took approximately 30 µkg of total protein fraction. Samples were mixed with 6x Laemmli buffer with mercaptoethanol, and heated at 95°C for 5 min. Protein electrophoresis was performed for 40 minutes at a constant voltage of 50 V, and then 60 minutes at a constant voltage of 120 V. Then samples were transferred to the Immun-Blot PVDF membrane (Bio-Rad, USA) for 1 h at a constant current of 175 mA. Nonspecific binding was blocked by 5% nonfat-dried milk, dissolved in PBS with 0.2% of Tween20, at 4°C for 90 min. The same solution was used for the preparation of the working solutions of the antibodies. Membrane was then incubated with the primary antibodies for PKCζ C-terminal domain (1:5000; Cat.#SAB4502380, Sigma) for 90 min at room temperature at constant shaking, and then washed four times in PBS with 0.2% of Tween20. The same protocol was used for incubation with the secondary goat anti-rabbit antibodies conjugated with horseradish peroxidase (1:3000; Cat.#172-1019, Bio-Rad, USA). Antigen–antibody complexes were visualized using Clarity Western ECL Substrate reagent (Bio-Rad, USA). The signals were

detected using ChemiDoc Touch Imaging system (Bio-Rad, USA) at parameters for Stain-Free gels. The pictures were processed using ImageLab v.5.2 software (Bio-Rad, USA).

Supplementary Figures

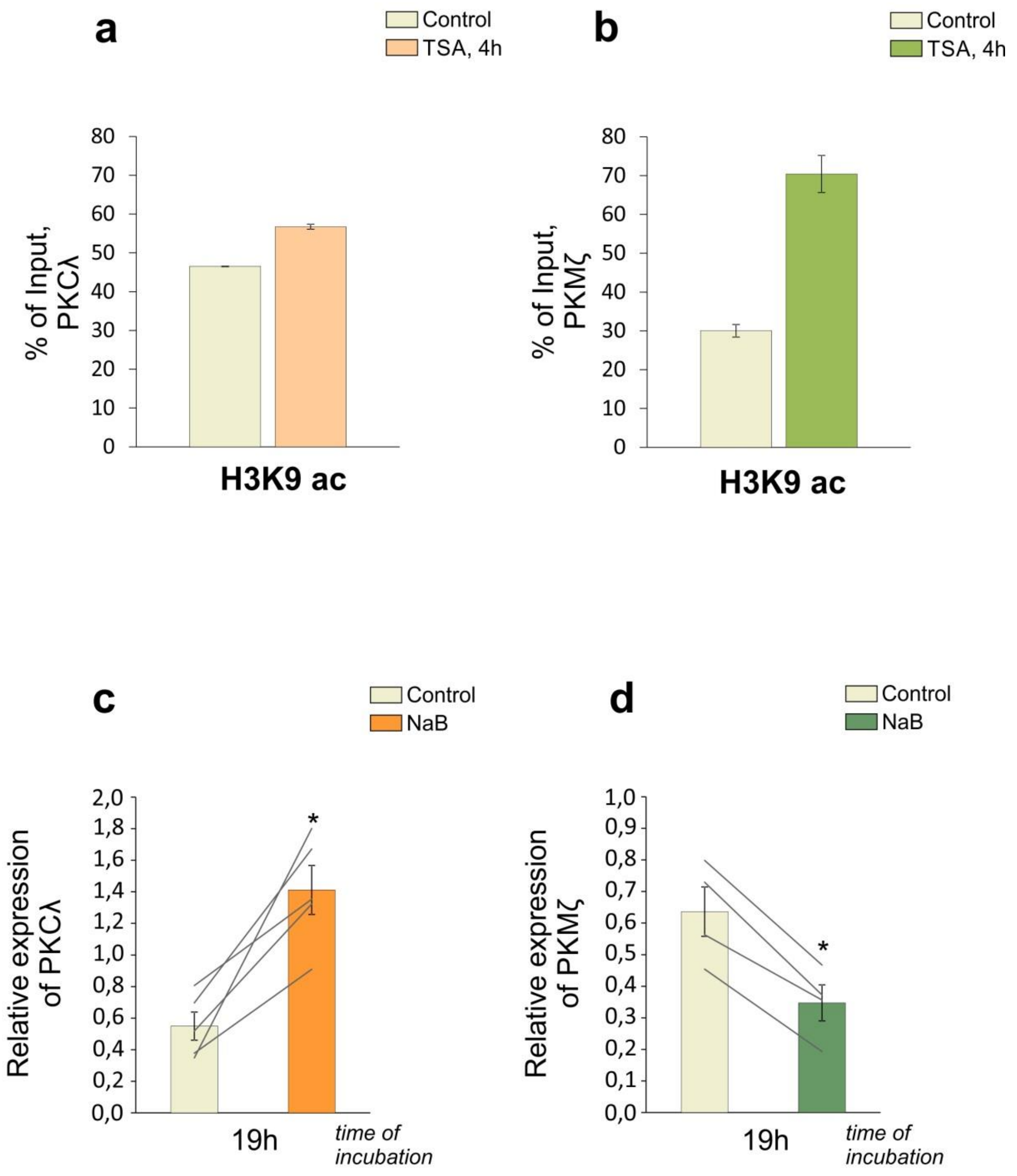

Supplementary Fig.S1. HDAC inhibitors induced acetylation of histones in promoter regions of aPKC genes, and influenced the expression of PKCλ and PKMζ. **(a)** Incubation of cultures with TSA for 4 h slightly stimulated histone acetylation in promoter region of *Prkci* gene [n=2/group], and **(b)** Considerably increased histone acetylation in downstream promoter of *Prkcz* gene [n=2/group]. **(c)** Nonselective HDAC inhibitor sodium butyrate (NaB, 5mM) induced upregulation of PKCλ expression in cultured cortical neurons [$F_{(1,8)}$ = 23,148; p=0,001; n=5/group]. **(d)** Nonselective HDAC inhibitor sodium butyrate (NaB, 5mM) produced changes in expression of PKMζ in cultured cortical neurons [$F_{(1,6)}$ = 8,911; p=0,024; n=4/group]. Each line represents the changes of control relative experimental cultures in individual experiment. * - significant differences

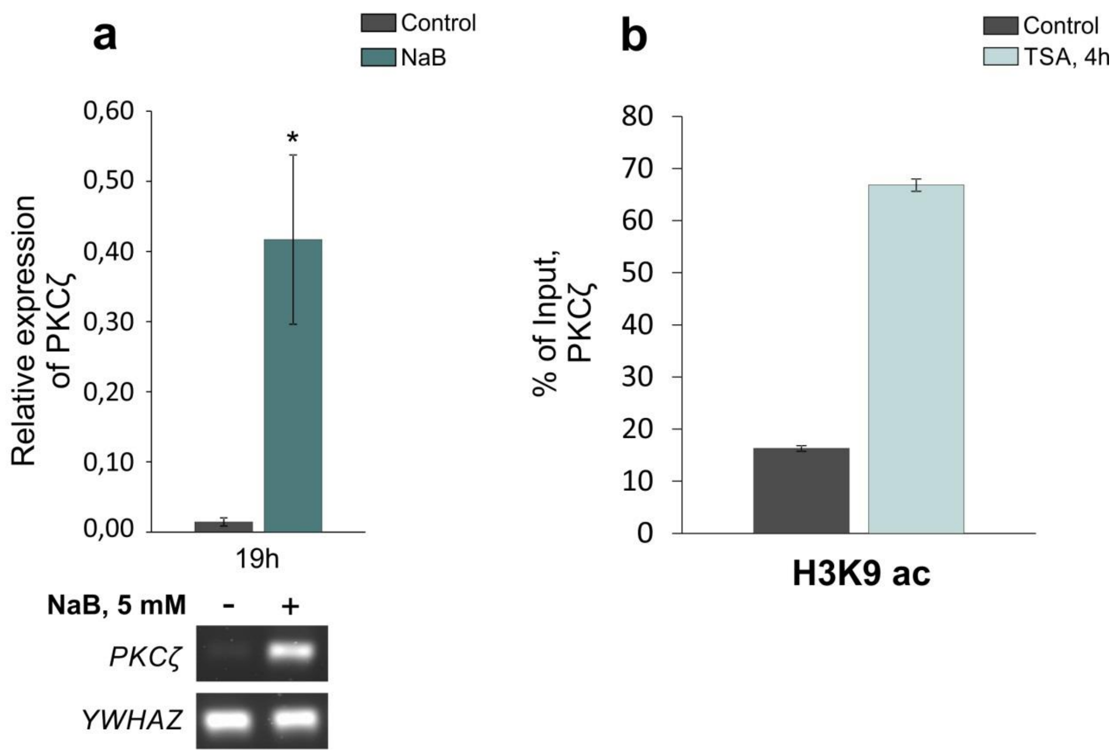

Supplementary Fig.S2. HDAC inhibitors stimulated the expression of PKCζ, and induced acetylation of histones in upstream promoter of *Prkcz* gene. **(a)** Sodium butyrate (NaB, 5 mM) induced upregulation of PKCζ expression in cultured cortical neurons [KW: H (1, N= 9) =6,0 p =,0143; *p=0,007; n=4-5/group]. Representative experiment below demonstrates the stimulation of PKCζ expression, caused by NaB. **(b)** Incubation of cultures with TSA for 4 h considerably increased histone acetylation in upstream promoter of *Prkcz* gene [n=2/group],

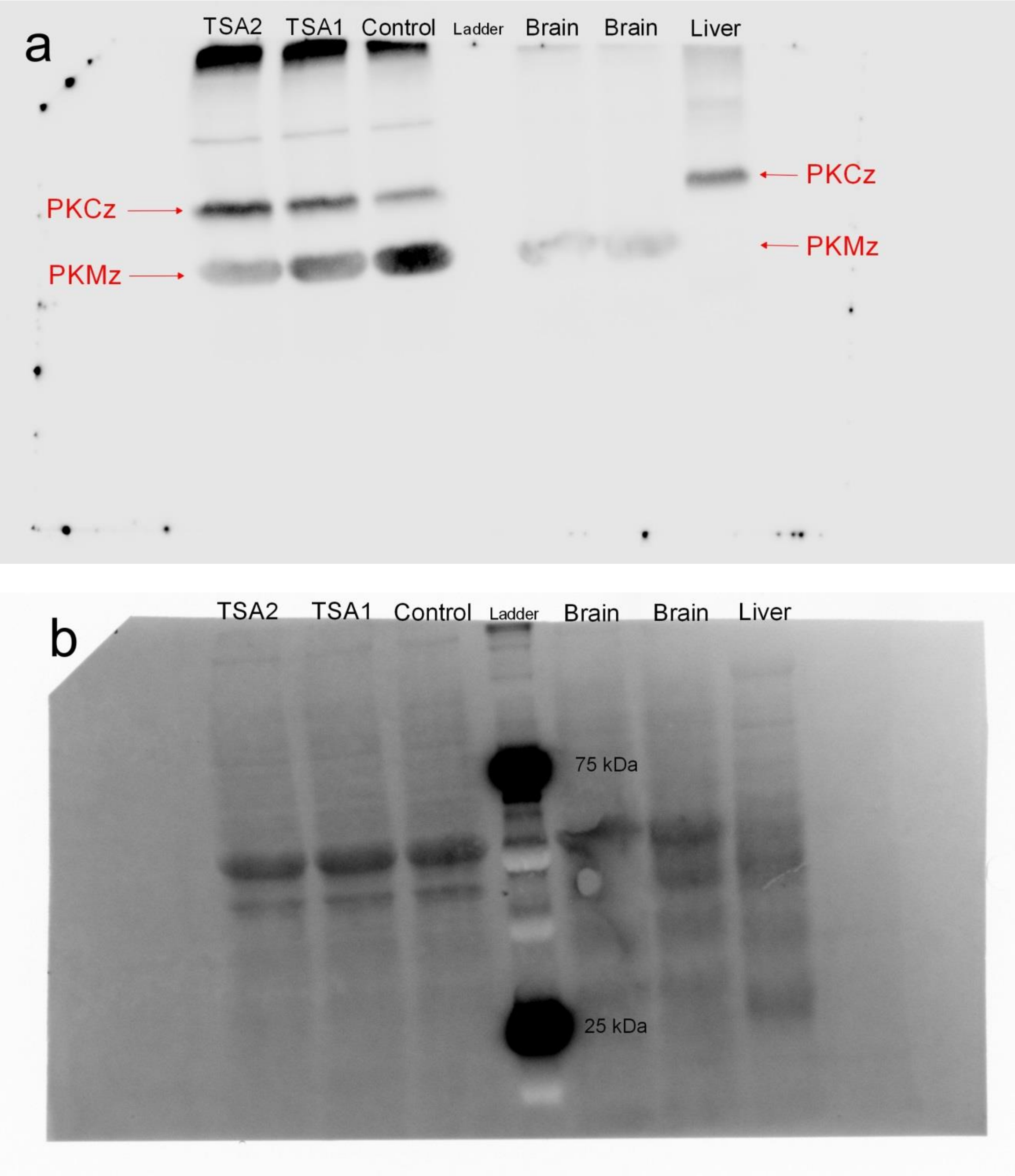

Supplementary Fig.S3. Continuous incubation of cortical cultures with TSA (19 h) influenced the protein levels of PKMζ and PKCζ isoforms. **(a)** Pilot experiment demonstrates the TSA-triggered stimulation of PKCζ expression and downregulation of PKMζ as compared to control sample [n=1-2/group]. The brain and liver samples were used for functional verification of lines, corresponding to brain-enriched PKMζ and peripheral PKCζ products. **(b)** Figure illustrates the approximate quantities of total proteins loaded into each well. The similar amounts were present in the wells with control and TSA-treated samples.

The supplementary figures S4-S7 were not scored for the main results and served as the illustrative experiments.

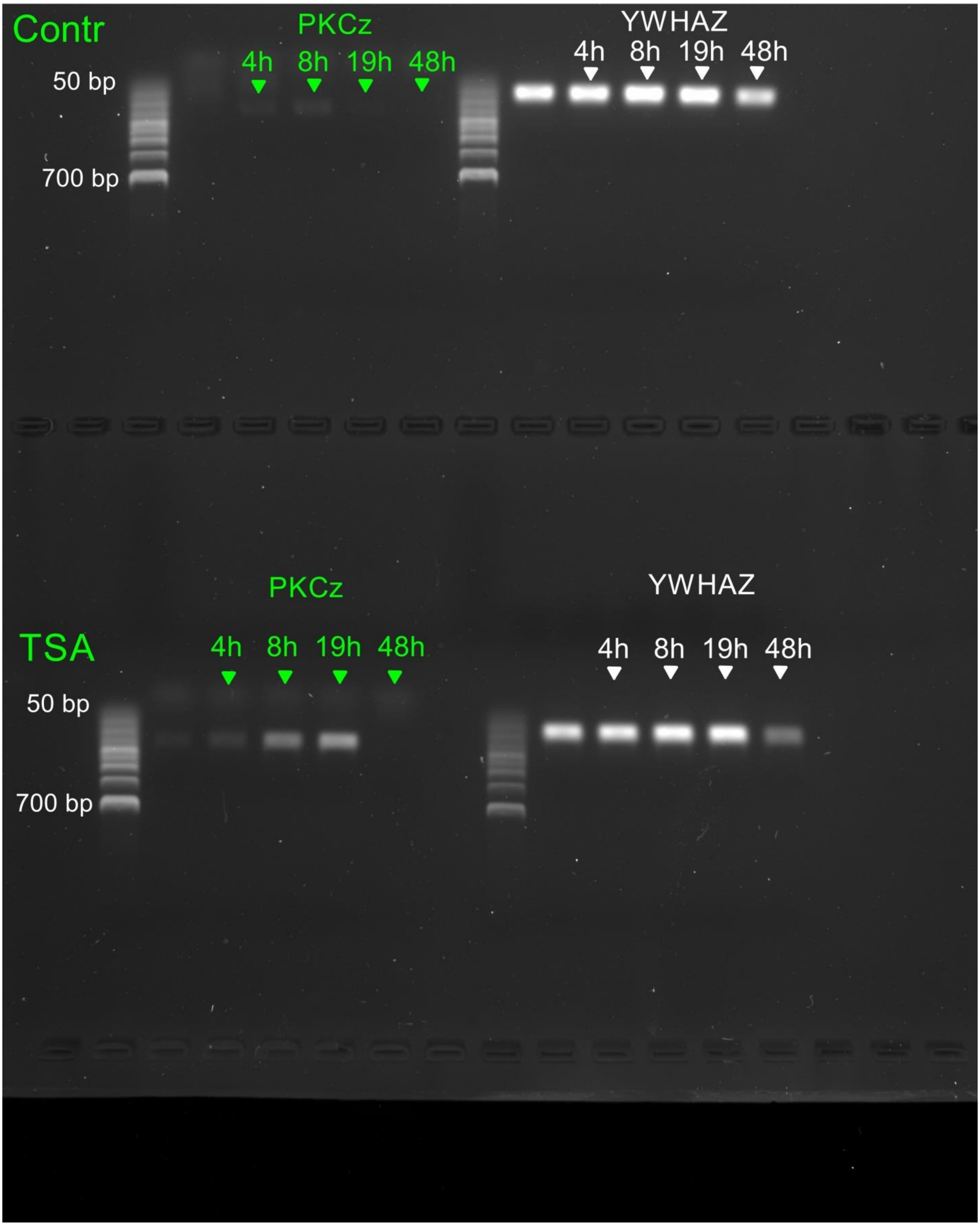

Supplementary Fig.S4. Dynamic changes of PKCζ expression in control cultures and cultures incubated with trichostatin A (TSA, 100 nM) for indicated time (hours). The full-length gel illustrates the representative experiment corresponding to Fig.3a.

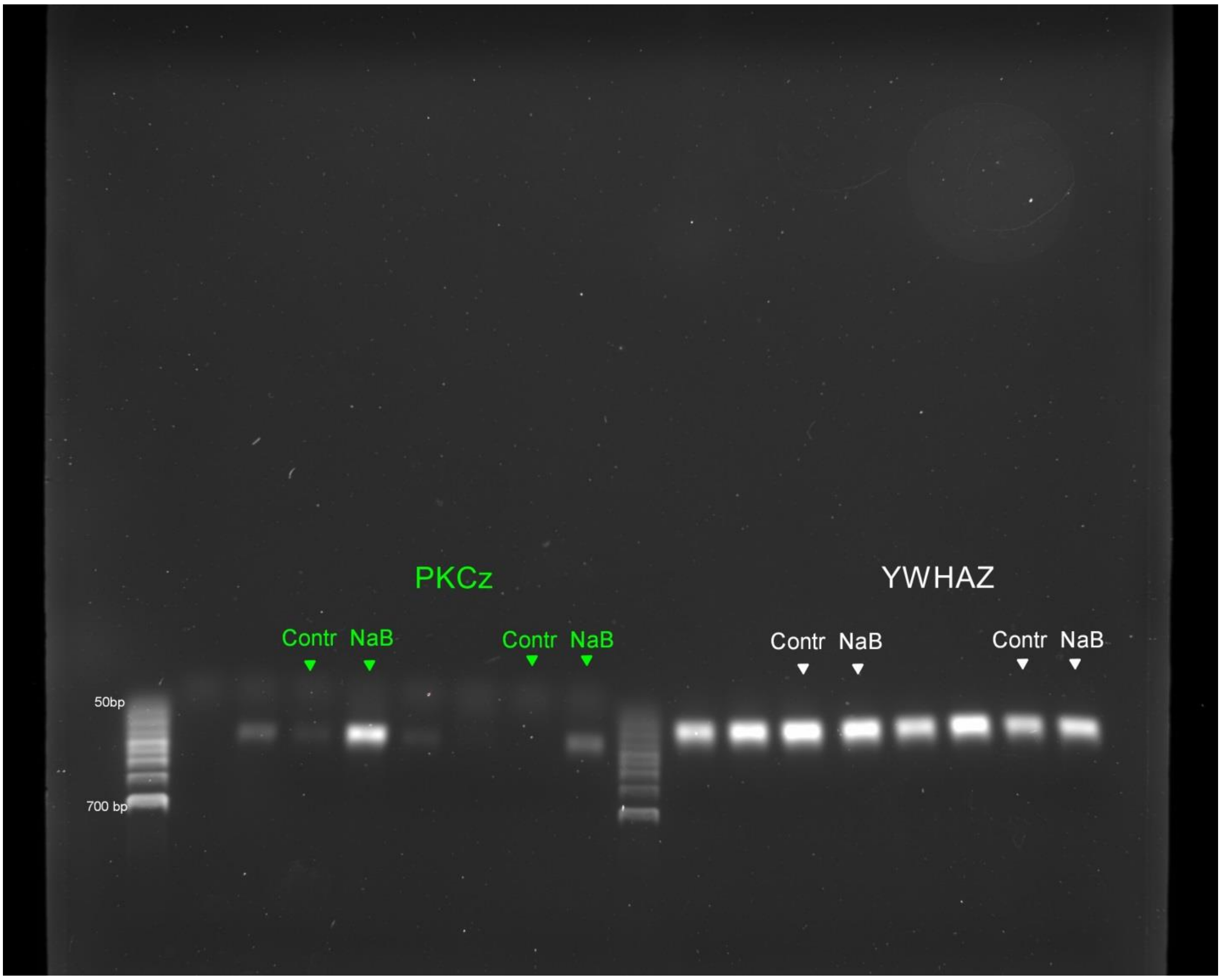

Supplementary Fig.S5. Sodium butyrate (NaB, 5 mM) stimulated the expression of PKCζ. The full-length gel illustrates the representative experiment corresponding to Supplementary Fig.S2a.

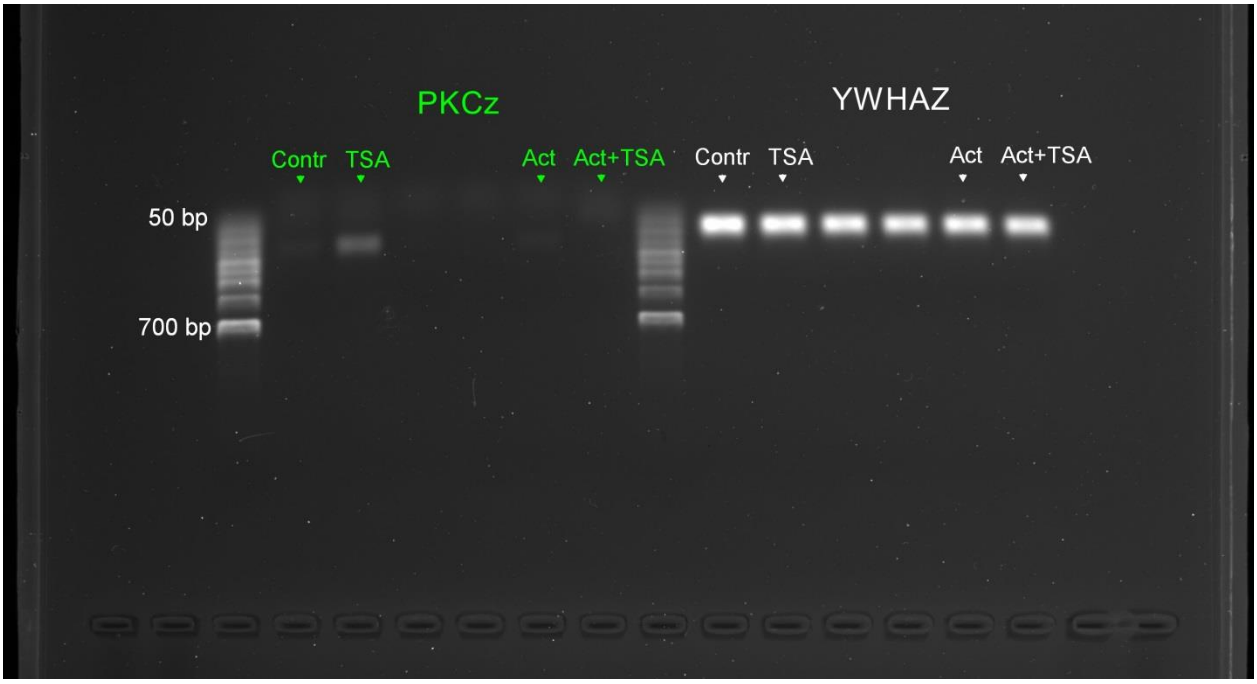

Supplementary Fig.S6. Transcriptional blockade with actinomycin D (ActD, 200 nM) prevented TSA-induced changes in mRNA levels of PKCζ. The full-length gel illustrates the representative experiment corresponding to Fig.4c.

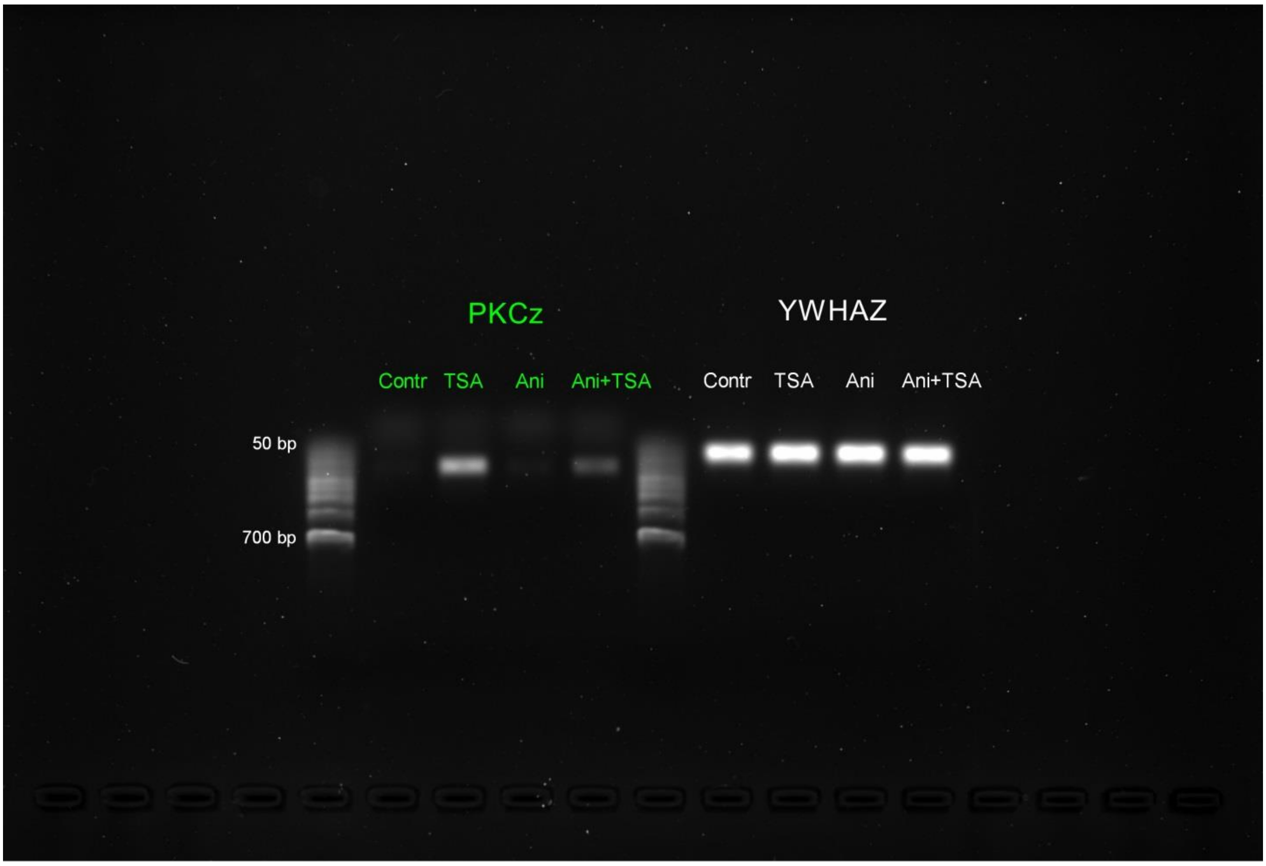

Supplementary Fig.S7. Anisomycin (Ani, 10 μM) significantly alleviated epigenetically-driven upregulation of PKCζ expression. The full-length gel illustrates the representative experiment corresponding to Fig.5c.
